# Association of Adipose Tissue and Adipokines with Development of Obesity-Induced Liver Cancer

**DOI:** 10.3390/ijms22042163

**Published:** 2021-02-22

**Authors:** Yetirajam Rajesh, Devanand Sarkar

**Affiliations:** 1 Department of Human and Molecular Genetics, Virginia Commonwealth University, Richmond, VA 23298, USA; Rajesh.Yetirajam@vcuhealth.org; 2 Massey Cancer Center, Department of Human and Molecular Genetics, VCU Institute of Molecular Medicine (VIMM), Virginia Commonwealth University, Richmond, VA 23298, USA

**Keywords:** adipose tissue, adiponectin, adipokines, leptin, NAFLD/NASH, HCC, therapeutic targets

## Abstract

Obesity is rapidly dispersing all around the world and is closely associated with a high risk of metabolic diseases such as insulin resistance, dyslipidemia, and nonalcoholic fatty liver disease (NAFLD), leading to carcinogenesis, especially hepatocellular carcinoma (HCC). It results from an imbalance between food intake and energy expenditure, leading to an excessive accumulation of adipose tissue (AT). Adipocytes play a substantial role in the tumor microenvironment through the secretion of several adipokines, affecting cancer progression, metastasis, and chemoresistance via diverse signaling pathways. AT is considered an endocrine organ owing to its ability to secrete adipokines, such as leptin, adiponectin, resistin, and a plethora of inflammatory cytokines, which modulate insulin sensitivity and trigger chronic low-grade inflammation in different organs. Even though the precise mechanisms are still unfolding, it is now established that the dysregulated secretion of adipokines by AT contributes to the development of obesity-related metabolic disorders. This review focuses on several obesity-associated adipokines and their impact on obesity-related metabolic diseases, subsequent metabolic complications, and progression to HCC, as well as their role as potential therapeutic targets. The field is rapidly developing, and further research is still required to fully understand the underlying mechanisms for the metabolic actions of adipokines and their role in obesity-associated HCC.

## 1. Introduction

Obesity is one of the most severe health disorders prevailing worldwide. Its prevalence has been increasing at an alarming rate for the last few decades. Obesity is defined as a body mass index (BMI) equal to or higher than 30 kg/m^2^. Obesity often leads to a series of medical disorders, including metabolic syndrome and nonalcoholic fatty liver disease (NAFLD), a spectrum including nonalcoholic steatohepatitis (NASH) [1]. It has also been recognized epidemiologically and clinically as a major risk factor for liver cancer. Because of its much wider spread and higher prevalence, obesity serves as a large contributor to overall hepatocellular carcinoma (HCC) [2]. The precise mechanisms underlying the obesity–liver cancer link are not yet completely unraveled. In general, obesity results from an impaired balance between calorie intake and energy expenditure, ultimately leading to the excessive accumulation of adipose tissue (AT). AT is not only recognized as a reservoir for excess energy derived from food but also as an endocrine organ. It produces adipocytokines or adipokines that trigger chronic low-grade inflammation in several organs of the body. It has been suggested that excessive AT and AT dysfunction dysregulate adipokine secretion, contributing to a variety of pathological processes, resulting in obesity-related liver cancer [3,4,5,6].

Broadly, AT is categorized into brown adipose tissue (BAT) and white adipose tissue (WAT). In the adult human body, the prevalence of BAT is minimal, only ~50 g in comparison to kilograms of WAT. Much evidence emphasizes that WAT is a metabolically active secretory endocrine organ [7,8]. It comprises different cells producing adipokines and cytokines [9]. Adipocytes tend to divide and give rise to new adipocytes upon stimulation and activation. The new white adipocytes increase or decrease in volume throughout their lifetime until the death of an individual [10]. 

WAT provides most of the total body fat, which is a source of free fatty acids (FFAs). FFAs are employed as substrates to generate energy via the oxidative phosphorylation of high-energy ATP bonds [11]. WAT is dispersed in the intra-abdominal region (omentum, intestines, and perirenal) and the subcutaneous region (buttocks, thighs, and abdomen) [11]. Two key functions of WAT are the regulation of metabolism and inflammation. It controls metabolism through energy homeostasis, adipocyte differentiation, and insulin sensitivity [12,13]. It regulates inflammation by the generation of pro- and anti-inflammatory molecules and the activation of metabolic and immune signaling [12,13]. Unfortunately, the excessive accumulation of WAT in body sites gives rise to obesity and obesity-associated diseases. In particular, WAT, deposited in the upper parts of the body, known as android obesity/central obesity, represents a risk factor for inflammatory pathologies [14]. Excess WAT deposited in the lower parts of the body give rise to gynoid obesity, with no metabolic issues [11,14]. Several theories have considered the underlying different distributions of WAT and its association with metabolic and inflammatory complications, including two theories that are not mutually exclusive. The first theory is based on the anatomical aspects of central obesity and its ability to drain FFAs and inflammatory mediators into the portal circulation, where they specifically act on hepatic cells, affecting the metabolism [11]. The second theory deals with the biological properties of WAT cells causing major/minor risks for developing metabolic and inflammatory complications [15]. Clinically, differential expression in several genes in different deposits of the body’s WAT has been reported [16,17]. The different types of cells that constitute WAT are mature adipocytes, preadipocytes, fibroblasts, endothelial cells, and macrophages [11,14,18]. Adipocytes, preadipocytes, and macrophages possess metabolic and inflammatory functions that cause WAT to release signaling molecules in WAT itself or other tissues at paracrine or endocrine levels [12,13,18,19]. Low-grade chronic obesity-related inflammation is specifically determined by macrophages that mediate circulating levels of specific inflammatory molecules [12,14,18,20,21]. Unlike WAT, BAT consists of a small number of fat cells with a rich vascular supply, an excess of mitochondrial chromogens, and energy expenditure from nonoxidative phosphorylation [22]. BAT has been highlighted as a potential target for pharmacological and genetic manipulation to combat obesity [23]. 

It is necessary to understand the process of energy homeostasis to interrogate the potential mechanisms involved in energy-rich conditions during overnutrition. The dynamic process of fat remodeling ensures adequate body fat and energy homeostasis without excess weight gain or loss. A positive energy balance with weight gain is dependent on conditions of increased appetite and food intake. Contrastingly, satiety determines a negative energy balance and weight loss by limiting food consumption. This whole phenomenon is under hypothalamic neuropeptide regulation of appetite and satiety [22]. Depending on physiological needs, a balance in energy homeostasis is achieved by the central and autonomic nervous system regulating energy expenditure [24]. Henceforth, more energy expenditure leads to WAT lipolysis and the augmentation of FFAs, whereas less energy expenditure favors an increase in fat storage [22]. The parasympathetic nervous system (PNS) enables fat deposition and a reduction in peripheral energy use [25]. The sympathetic nervous system (SNS) stimulates lipolysis by facilitating the release of FFAs for higher energy expenditure [26]. SNS innervations and β3 adrenergic receptors are predominant in both BAT and WAT. In BAT, uncoupled oxidative phosphorylation elevates heat production during cold-induced SNS activity [26,27]. In response to cold, high SNS activity stimulates WAT, which subsequently elevates thermogenesis through the oxidative phosphorylation of FFA in liver, muscle, and fat cells. This process is increased during obesity [26]. Heat-intolerant obese individuals tend to dissipate less heat from WAT and BAT, and at the same time, BAT mass and function are significantly reduced [22]. Thus, in general, obesity is induced by high calorie intake and reduced energy expenditure.

## 2. Lipid Metabolism in the Liver Regulated by Adipokines

Hepatic cells are mostly affected by ectopic lipid accumulation since the liver is the master regulator of systemic lipid and glucose accumulation. Consequently, NAFLD and NASH are the most common liver disorders, with up to a 90% prevalence in the obese population [28]. NAFLD and NASH are strongly associated with insulin resistance, progressing to cirrhosis and eventually to HCC, highlighting that excess lipid deposition in the liver leads to severe pathological consequences [29,30].

Albumin- and lipoprotein (LP)-mediated canonical lipid transport mechanisms regulate the mobility of lipids between tissues, such as the flux of lipids from AT to the liver and skeletal muscle or from the liver to AT and skeletal muscle. The impact of AT lipolysis on hepatic lipid accumulation has been illustrated by several studies, where fasting mice for 16 h induced nearly a 10-fold increase in hepatic lipid accumulation [31]. Several studies have identified mechanistic links, providing the basis for the AT–liver communication axis regulating systemic metabolic homeostasis and facilitating the understanding of the role of lipid transport, synthesis, and utilization in hepatic lipid metabolism and deposition [32,33,34]. Thus, it is important to understand the crosstalk between adipose tissue and the liver from a lipid-centric perspective in the maintenance of systemic energy homeostasis. Two AT-secreted factors, adiponectin (ADP) and leptin, play a seminal role in regulating lipid metabolism in the liver.

### 2.1. Adiponectin (ADP)

Serum ADP is found to be reduced in obese, insulin-resistant individuals [35]. ADP overexpression in obese conditions has been reported to prevent high-fat diet (HFD)-induced lipid accumulation [36]. Moreover, ADP knockout in leptin-deficient (ob/ob) obese mice augments hepatosteatosis [32]. The subtypes of adiponectin (ADP) receptors that are expressed in the liver are adipoR1 and adipoR2 [37]. ADP reduces hepatic lipogenesis and elevates fatty acid (FA) β-oxidation via the adipoR1-mediated activation of 5′-Adenosine Monophosphate-Activated Protein Kinase (AMPK) and Peroxisome Proliferators-Activated Receptor α (PPARα) [34,38,39]. AMPK inhibits lipogenesis by phosphorylating Acetyl-CoA Carboxylase-1 (ACC-1), reducing ACC-1 activity and malonyl CoA production thereby releasing the inhibition of Carnitine Palmitoyl Transferase 1 (CPT1) activity and stimulating the transport of FA into mitochondria for undergoing β-oxidation. At the transcriptional level, ADP induces the LKB-AMPK pathway via adipoR1, reducing the expression of hepatic lipogenesis and cholesterol synthesis genes by suppressing Sterol Regulatory Element Binding Protein 1c (SREBP1c) expression [34]. SREBP1c stimulates the expression of ACC-1 along with nuclear receptor Liver X Receptor (LXR) in the liver [40]. Hence, SREBP1c knockout prevents lipid accumulation in HFD-fed and leptin-deficient mice [41].

Independent of AMPK activation, the modulation of ceramide metabolism has been attributed to confer the pleiotropic functions of ADP, promoting insulin sensitivity, decreasing inflammation, and supporting cell survival [42]. ADP enhances ceramide catabolism by stimulating ceramidase activity through AdipoR1 and AdipoR2, resulting in the formation of the antiapoptotic molecule sphingosine-1-phosphate (S1P) [42]. The overexpression of acid ceramidase in HFD-fed mice prevents hepatic lipid accumulation and aids insulin sensitivity [33]. As such, ceramidase activity induced by ADP signaling might be key to its therapeutic efficacy. The metabolic effects of fibroblast growth factor 21 (FGF21), secreted by adipose tissue, the liver, and skeletal muscle, are facilitated by ADP. FGF21 has been reported to restore euglycemia, ameliorate hyperlipidemia, and reduce fat mass in obese models [43,44]. It was documented that FGF21 reduces ceramide accumulation in the liver and stimulates ADP secretion. Indeed, genetic ablation of ADP in leptin-deficient (ob/ob) and diet-induced obese mice prevented FGF21 from exerting its beneficial effects, indicating that FGF21 depends on ADP for lipid-lowering effects in obese mice [32].

### 2.2. Leptin

Leptin exhibits catabolic action in the adipocytes, preventing lipogenesis and activating β-oxidation of FA in the liver. Leptin receptors are abundantly expressed in the hepatic cells, and their expression is upregulated in response to leptin stimuli and short-term fasting [45]. Leptin-deficient ob/ob mice and leptin-receptor-deficient db/db mice have been reported to present with hypertriglyceridemia, hypercholesterolemia [46], and diminished lipid tolerance [47], ultimately leading to steatosis [48]. A study involving liver-specific leptin receptor knockout mice revealed reduced circulating levels of apolipoprotein B, high very-low-density lipoprotein triglyceride (VLDL TG), and hepatic lipoprotein lipase activity, suggesting changes in TG incorporation into VLDL or abnormal lipoprotein remodeling in the plasma [49]. Leptin also elevates hepatic FA oxidation and reduces de novo lipogenesis through ACC-1 phosphorylation [50].

Additionally, ADP and leptin exert anti-inflammatory actions on hepatic cells, aiding in the prevention of the transition from NAFLD to NASH. Antifibrotic and anti-inflammatory activity of ADP reduces hepatic fibrosis in steatohepatitis models [51,52]. Likewise, recombinant leptin and metreleptin (leptin analog), administered for lipodystrophy and congenital leptin deficiency, have been reported to reverse hepatic lipid accumulation and reduce the consequences of NASH [53]. Metreleptin is currently under clinical trial (NCT01679197), showing potential hope for adipose-derived secretory products to combat obesity-associated hepatic disorders.

## 3. Role of AT and Adipokines in Hepatic Functioning

AT comprises major cell types—adipocytes, preadipocytes, endothelial, and immune cells. It stores excess energy as TG in the lipid droplets of adipocytes through hyperplasia (increase in the number of adipocytes) or hypertrophy (size enlargement of adipocytes) [54]. The number of adipocytes is age-dependent, variable during childhood and adolescence, and constant during adulthood, irrespective of body weight (lean or obese) [55]. Elevated fat mass during adulthood primarily contributes to hypertrophy. A recent study has highlighted the hyperplasia of adipocytes in adulthood, where normal-weight adults expand only lower-body subcutaneous fat by increasing the number of adipocytes in response to overfeeding [56].

**Figure 1:**
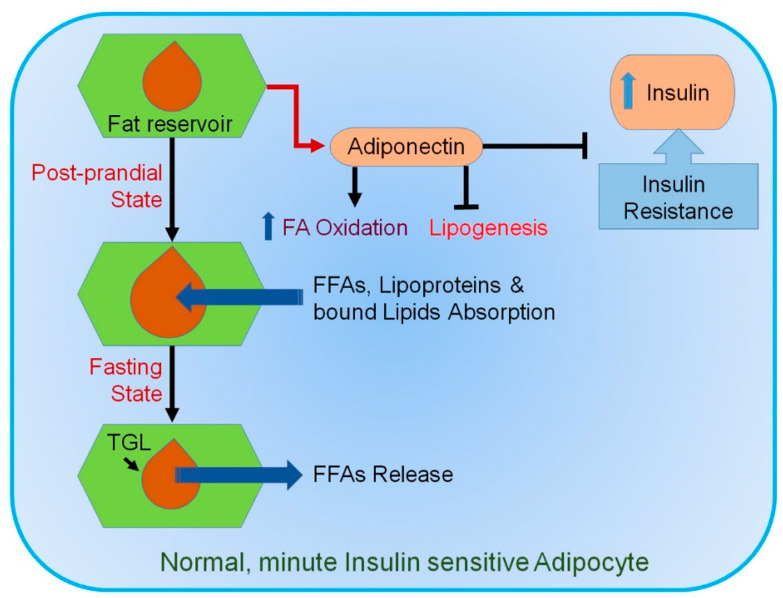
Mechanism involved in normal healthy adipocytes. In response to a sedentary lifestyle, food intake, and an increase in circulating insulin, small healthy adipocytes absorb different types of lipids. They tend to suppress lipogenesis and secrete adiponectin (ADP). ADP promotes insulin sensitivity and fatty acid (FA) oxidation. During fasting, triglyceride lipases (TGLs) become activated, releasing free fatty acids (FFAs). Blue upward arrow indicates an increase.

While obesity is allied with metabolic disorders, hypertrophy-mediated AT dysfunction plays a significant role in the development of insulin resistance (IR) [57]. During energy requirement, TGs stored in adipocytes are mobilized through lipolysis and release FFA, which subsequently move to other tissues to be employed as an energy source, as displayed in Figure 1.

FFAs play a vital role in the development of obesity-associated metabolic disorders. In obesity, FFAs enter the liver through the portal circulation, and high hepatic FFAs induce lipid synthesis, gluconeogenesis, and IR in the liver [58]. High levels of circulating FFAs leading to peripheral IR have been reported in both in vivo studies and in humans [58,59]. Additionally, FFAs modulate the inflammation of AT contributing to obesity-related metabolic complications by serving as ligands for the TLR4 complex [60] and stimulating cytokine production by macrophages [61]. Many studies also indicate a direct relationship between FFA release from AT and obesity-associated metabolic complications.

AT secretes multiple adipokines such as chemokines, cytokines, and hormones. Many of them are involved in energy homeostasis and inflammation. In obesity, adipocytes develop obesity-induced inflammation under the influence of a high secretion of chemokines and cytokines [62,63]. Many of them, such as MCP-1, TNF-α, IL-1, IL-6, and IL-8, promote IR [64,65,66,67]. Moreover, macrophages from AT are correlated with adipocyte size and body mass and proinflammatory cytokines [68]. Along with a high number of macrophages in AT, obesity stimulates a phenotypic switch from an anti-inflammatory M2 polarization state to a proinflammatory M1 polarization state [69]. The accumulated M1 macrophages in AT secrete a variety of proinflammatory cytokines and chemokines, contributing to obesity-linked IR [70]. On the other hand, M2-polarized macrophages facilitate AT remodeling, involving the clearance of dead adipocytes and the enrolment and differentiation of adipocyte progenitors [71]. The decreased expression of inflammatory cytokines in AT and increased insulin sensitivity in obese models have been observed by reduced adipose macrophage infiltration or macrophage removal [72,73]. Moreover, in obese conditions, weight loss reduces macrophage infiltration and proinflammatory gene expression in adipose tissue [74,75]. In obesity, proinflammatory immune cells, such as interferon (IFN)-γ^+^T helper type 1 cells and CD8^+^T cells, are elevated [76], with reduced secretion of insulin-sensitizing adiponectin in AT [77].

WAT produces and secretes adipocytokines or adipokines, but in hypertrophy, a change in protein synthesis leads to the production of inflammatory proteins, such as cytokines [78,79,80]. In general, adipokines, such as TNF-α, IL-6, and PAI-1, are proinflammatory, whereas adiponectin is anti-inflammatory, antidiabetic, cardioprotective, and antitumorigenic [81,82]. In obesity, dysfunctional AT releases higher levels of proinflammatory factors and reduced adiponectin [83]. This difference in the pathophysiology of adipocytes aids in understanding the association between obesity, IR, metabolic syndrome, atherosclerosis, and cancer [84,85]. A close association exists between the production of inflammatory proteins and the level of hypertrophy of adipocytes [61,86]. A summary of key proteins secreted by adipocytes normally and during hypertrophy is shown in Table 1.

Adipokines comprise heterogeneous proteins, which, functionally, are polyvalent molecules involved in physiological and pathological processes modulating the sensitivity of peripheral tissues to insulin, regulating appetite, homeostasis, energy expenditure, and glucose and lipid metabolism [87,88]. Additionally, they are strongly related to immunity and inflammation [89,90]. WAT plays a crucial role in controlling physiological and pathological processes (metabolism and energy homeostasis) [91,92]. It communicates directly with peripheral tissues, particularly skeletal muscle, through various adipokines. Intense crosstalk exists between WAT and the brain through leptin and SNS [93,94].

Plasma TNF-α levels are higher in hyperlipidemic patients and are positively correlated with VLDL TG concentrations [95], which are associated with hepatic TG synthesis and the secretion and inhibition of LPL [96,97]. Additionally, TNF-α promotes the synthesis of hepatic apolipoprotein (apo) B100-VLDL by impairing hepatic insulin signaling [98]. Similarly, IL-6 is also related to hypertriglyceridemia. Hypertriglyceridemia and upregulated serum TG subjects produce high levels of IL-6 and TNF-α [99,100]. Interestingly, high anti-inflammatory cytokine levels, such as IL-10, are also related to high-plasma TG levels [101]. Other proinflammatory cytokines, IL-6, IL-1, IFN-α, and IFN-γ, promote TG synthesis in HepG2 cells [102], stimulate lipolysis in adipocytes [103,104], and reduce LPL activity in vivo and in vitro [105,106,107]. Many studies also report that TNF-α and IL-6 are negatively related to serum HDL-cholesterol levels in healthy individuals and cardiovascular patients [108,109], and IL-10 is positively related to plasma HDL-cholesterol levels [101]. There are also reports of the TNF-α-, IL-6-, and IL-1-mediated reduced expression of apo A1 in hepatic cells and plasma [105]. Proinflammatory cytokines upregulate circulating total cholesterol and LDL-cholesterol levels by activating cholesterol synthesis [105,110], and higher IL-10 levels are negatively correlated with increased levels of total cholesterol and LDL [101]. TNF-α, TGF-β, or IL-1 promote lipoprotein uptake by LDL and scavenger receptors and inhibit ABCA1-mediated cholesterol efflux to HDL, which contributes to lipid deposition and the formation of foam cells [111,112,113]. Additionally, in the absence of extracellular FAs, TNF-α increases the secretion of apo B in hepatocytes [114]. Cytokine treatment also stimulates the hepatic production and secretion of phospholipase A2 [115].

## 4. Adipokine-Mediated Modulation of Cell Death and Survival in Hepatocytes

### 4.1. Prosurvival Effects of ADP

ADP contributes toward effects on metabolic disorders and the development and progression of cancer. It has been reported to suppress tumor growth and inversely associate with the incidence of cancer [116]. ADP modulates hepatic physiology by controlling cell death and the survival of primary hepatic cells [117]. ADP protects hepatic cells from iron-overload-induced hepatic injury by Heme Oxygenase 1 (HO1) induction and PPAR-α activation [118] and suppresses ethanol-induced apoptotic cell death through Nrf-2 signaling and HO1 induction [119]. Hepatic apoptosis is considered to be an early feature of alcoholic liver disorder. ADP protects hepatic cells by inhibiting CD95 upregulation [120] and ameliorates AMPK/eNOS signaling-mediated hepatic apoptosis and inflammation [121], thereby providing a prominent therapeutic option for alcohol-induced liver injury. It has been shown to induce autophagy and promote cell survival by enhancing autophagy-related gene expression through the AMPK-dependent nuclear translocation of FoxO3A [119]. ADP-mediated autophagy digests Bax and prevents caspase activation [119], and adipoR1 signaling enhances the expression of chemokine-like receptor 1 and CXCL8 [122,123] and attenuates ceramide accumulation. AdipoR1 and R2 signaling increases ceramidase activity and the formation of S1P, which contributes to the prosurvival effect of ADP in the liver [42].

Hepatocyte Growth Factor (HGF) and FGF-2 produced by hepatic stellate cells (HSCs) promote hepatic fibrosis [124,125]. ADP induces apoptosis and inhibits HSCs, affects the survival of Kupffer cells, and promotes conversion to the M2 phenotype for releasing mediators that stimulate M1 macrophage apoptosis [126]. Additionally, it induces apoptosis of murine macrophage cells through reactive oxygen species (ROS) production from reduced NADPH oxidase and iNOS [127,128], affects activated and/or quiescent status of HSCs, prevents the proliferation of HSCs, and induces apoptosis of activated HSCs [129]. ADP induces the expression of p53 and Bax and inhibits the GSK-3β mediated prosurvival pathway, thereby inhibiting the activity of β-catenin or cyclin D1.

### 4.2. Effect of Leptin on Proliferative Potential of Hepatic Cells

Leptin mainly induces the proliferation and activation of HSCs without exerting significant proliferation/survival effects on hepatocytes and Kupffer cells. It is predominantly produced from AT and activated HSCs, impairing hepatic fibrosis resolution [129], inhibiting TRAIL-induced apoptosis, and suppressing FasL-induced apoptosis in HSCs [130]. Leptin also promotes liver fibrosis by inducing Transforming Growth Factor β (TGF-β) and connective tissue growth factor in HSCs [131]. The leptin receptors expressed in hepatocytes are involved in ROS production through NADPH oxidase-dependent signaling, although it does not significantly affect the function of primary mouse hepatocytes [132]. However, leptin has been reported to stimulate proliferation and suppress apoptosis of hepatic cancer cells. Its plasma level is higher in obesity, and its role in cancer progression suggests an underlying mechanism for cancer development in obese individuals [133]. It induces cell cycle progression and elevates the number of cells in the S and G2-M phase, enhancing DNA synthesis and mitotic activities [134]. The inhibition of the ER stress-associated apoptotic pathway contributes to a leptin-induced increase in liver cancer cells [135]. The induction of methionine adenosyltransferases confers the mitogenic property of leptin in liver cancer cells [136]. Leptin induces autophagy via a p53/Foxo3A axis, which, in turn, abrogates apoptosis of cancer cells [137].

Apoptosis in hepatic cells has been reported to mediate NAFLD/NASH and stimulate immune cells and hepatic stellate cells to produce inflammasomes and cytokines, leading to liver fibrosis [138]. These processes are usually accelerated by microbiota and abnormal glucose and lipid metabolism. In NASH, saturated FAs and mitochondrial cholesterol accumulation induce hepatic apoptosis, which involves the production of reactive oxygen species, oxidative stress, and endoplasmic reticulum stress [138]. Apoptotic events in NASH are caspase-dependent and are associated with mitochondrial membrane depolarization and cytochrome c release. Subsequently, the mitochondrial apoptotic pathways are activated through the activation of Bax and Bim, and c-Jun N-terminal kinase (JNK) plays an important role in mediating these events [139,140]. In the liver, MLK3 is one of the MAP3K proteins that mediate JNK activation during NASH [141]. Apoptosis inhibitors have been employed for NASH treatment, cirrhosis prevention, and HCC. A list of important apoptotic markers mediating NAFLD/NASH is shown in Table 2.

**Table 2:** Different apoptosis markers involved in nonalcoholic fatty liver disease (NAFLD)/nonalcoholic steatohepatitis (NASH).

Type	Subtype	Effect	Case	References
Caspases	Caspase 3 and 7	Strongly correlated with hepatocyte apoptosis	NASH	[142]
		Correlated with disease severity	NAFLD	
	Caspase 3 generated CK-18 fragments		NASH Predictor	[142,143]
	Caspase 9	Executes mitochondrial apoptosis pathway	NASH	[144]
	Caspase 2	Initiator caspase in lipid-induced cytotoxicity	NASH	[145]
Bcl-2	Bax		NAFLD, NASH	[146]
	Bcl-2	Promotes apoptosis by modifying the expression and function of Bcl-2 homology 3 (BH3) only protein Bim and PUMA	Hepatic lipoapoptosis	[147]
	Transglutaminase 2	Crosslinks and inactivates transcription factor Sp1, resulting in hepatic apoptosis	NASH	[148]
	Bid	Higher expression of HDMCP induces hepatocyte apoptosis triggered hepatic stellate cell activation.	NASH	[149]
JNK	Sh3bp5	Impaired respiration, ROS production, JNK activation, and apoptosis	NASH	[150]
	CREG	Interact with ASK1 and inactivate ASK1-JNK1 signaling	NAFLD	[151]

## 5. Putative Mechanisms for Obesity-Induced Liver Cancer

### 5.1. Function of Adipose-Tissue-Produced Adipokines in Insulin Resistance

Insulin resistance (IR), defined by decreased responsiveness to insulin and characterized by reduced glucose disposal in nonhepatic tissues, is one of the obesity-related disorders posing a major risk toward cancer development and is associated with poor prognosis [152]. IR patients exhibit high glucose and insulin levels due to reduced insulin sensitivity [153]. The dysfunction of adipocytes, either because of genetic factors or induced by HFD, plays an integral role in IR [154]. WAT synthesizes, esterifies, and stores TG in lipid droplets, and it is highly insulin-sensitive [155]. Insulin acts by binding to the insulin receptor (IR) with the activation of the PI3K/Akt pathway and increases glucose uptake, augments the utilization of glucose for the production of glycerol, and inhibits lipolysis by lipases [155]. In obesity, IR promoted by proinflammatory cytokines leads to the chronic low-grade inflammation of AT, thereby providing a microenvironment for tumorigenesis [156]. Hypoxic hypertrophic adipocytes have elevated levels of inflammatory cytokines (TNF-α, IL-1, and IL-6) and reduced ADP [157]. These cytokines can function either directly by deactivating insulin receptor substrate-1 (IRS1) via the phosphorylation of serine and threonine residues or decreased tyrosine phosphorylation [158,159,160,161] or indirectly, wherein upregulated FFA increases NF-kB, which is implicated in IR [162].

#### 5.1.1. Chemokines

In obesity, the expression of chemokines and their receptors is augmented in visceral and subcutaneous adipose tissue. Chemokines selectively recruit monocytes, neutrophils, and lymphocytes and induce chemotaxis [163]. C-C motif chemokine ligand 2/macrophage chemoattractant protein-1 (CCL2/MCP-1) regulates the migratory and infiltrative potential of monocytes/macrophages and initiates the inflammation of AT [164]. It is expressed by adipocytes and is correlated with adiposity. It has been reported that in AT, the overexpression of CCL2/MCP-1 elevates macrophage recruitment [165], whereas knockdown reduces the accumulation of proinflammatory macrophages, providing protection from IR and hepatic steatosis [68,165]. Interestingly, primary adipocytes show inherent immune function in which adipocyte-derived CCL2/MCP-1 stimulates inflammation and activates CD4+ T cells independently of macrophages/leukocytes in AT [166].

Other chemokines involved in AT macrophage infiltration and obesity-induced IR are CCL5, C-X-C motif chemokine ligand 5 (CXCL5), and CXCL14 [73,167,168]. In AT of obese individuals, multiple chemokines, CCL2, CCL3, CCL5, CCL7, CCL8, CCL11, CCL19, CXCL1, CXCL5, CXCL8, and CXCL10, and their receptors, CCR1, CCR2, CCR3, and CCR5, are highly expressed [163,168]. It has been shown that CXCL5, secreted from WAT-resident macrophages, interferes with insulin function by activating Jak2/STAT5/SOCS2 signaling, thus inducing IR, and treatment with anti-CXCL5 neutralizing antibody or antagonists of CXCL5 receptor CXCR2 provides protection in obese, IR mice [168].

#### 5.1.2. TNF-α

TNF-α is highly expressed in obese and IR individuals and is positively correlated with IR [169]. Indeed, TNF-α deletion improves insulin sensitivity in obese mice [170]. In healthy overweight individuals with metabolic syndrome and IR, the correlation between plasma TNF-α levels and IR is rather weak [64,171], and the chronic neutralization of TNF-α fails to improve the status of IR [172], suggesting an obesity-specific function of TNF-α. It has been also reported that TNF-α antagonist etanercept administration fails to improve insulin sensitivity in patients with metabolic syndromes [173], which might be explained by the compensatory role of other cytokines in the absence of TNF-α since various other proinflammatory cytokines, such as IL-1, and IL-6 secreted by AT, are involved in the disruption of insulin signaling [174].

#### 5.1.3. IL-6 and IL-18

IL-6 plays a crucial role in developing IR in obesity [175]. AT contributes to 10–35% of circulating IL-6 levels, and the hypertrophic enlargement of adipocytes results in high levels of IL-6 [176,177], which are positively correlated with IR [178]. IL-18 is a TNF-α-induced proinflammatory cytokine produced by AT in high levels in obese individuals, and its levels are reduced with weight loss [179]. The overexpression of IL-18 aggravates IR in rats [180]. Interestingly, the deletion of IL-18 or its receptor in mice induced hyperphagia, obesity, and IR because of increased food intake [181]. The lack of IL-18 in the liver caused the defective phosphorylation of STAT3, resulting in the enhanced expression of genes regulating gluconeogenesis contributing to hepatic IR. The intracerebral administration of recombinant IL-18 inhibited food intake and reversed hyperglycemia, indicating the tissue-specific function of IL-18 in AT and in the brain [181].

#### 5.1.4. Leptin

Leptin is profusely expressed in adipocytes regulating energy and glucose homeostasis, inhibiting appetite and food intake and stimulating energy expenditure [182]. A decrease in fat mass results in the lowering of plasma leptin levels, stimulating appetite and suppressing energy expenditure, while in obesity, increased fat mass results in increased leptin levels, suppressing appetite until body weight is decreased. Circulating leptin levels and leptin levels in AT are proportional to fat mass, but the level is rapidly decreased by fasting and increased by proinflammatory cytokines [183,184,185,186]. A bidirectional interaction exists between leptin and inflammation, in which proinflammatory cytokines elevate the synthesis and release of leptin, contributing to a chronic inflammatory state in obesity [187]. In the liver and skeletal muscle, leptin improves insulin sensitivity, but it exerts tissue-specific effects so that it inhibits adipocyte insulin signaling as an autocrine signal by abrogating insulin-induced MAPK activation, GSK3β phosphorylation, and insulin receptor tyrosine phosphorylation. Furthermore, indirectly through neuroendocrine pathways and in pancreatic β-cell, it impairs insulin secretion [188,189]. Leptin’s cytokine-like structure and its receptor, a member of the class I cytokine receptor (gp130) superfamily, promotes the production of proinflammatory (Th1) cytokines (IL-2 and IFN-γ) and inhibits the production of anti-inflammatory (Th2) cytokine (IL-4) by T cells/mononuclear cells, thus exerting an immunomodulatory effect [187,190].

#### 5.1.5. Resistin

Resistin is an adipokine promoting inflammation and IR [191]. Circulating levels of resistin are higher in obesity and are correlated with IR. Hyperresistinemia created by acute resistin infusion or stable resistin gene transfer causes IR in rodents, whereas its absence protects from diet-induced hyperglycemia as well as IR in ob/ob mice by increasing AMPK activity and decreasing the levels of gluconeogenic enzymes in the liver [192,193,194]. It inhibits multiple steps in insulin signaling, including IR and IRS-1 phosphorylation, PI3K activation, phosphatidylinositol triphosphate production, and the activation of Akt, by inducing the expression of an inhibitor of insulin signaling suppressor of cytokine signaling-3 (SOCS-3) in adipocytes and AT [195]. In rodents, resistin is produced exclusively from AT, whereas in humans, resistin is produced mainly by mononuclear cells, especially macrophages, and macrophage-derived human resistin exacerbates AT inflammation and IR [196,197].

#### 5.1.6. PAI-1

A primary inhibitor of fibrinolysis PAI-1 is synthesized in AT by adipocytes and stromal vascular cells, such as preadipocytes, fibroblasts, vascular endothelial cells, and immune cells. Its levels are high during obesity and IR [198,199]. The lack of PAI-1 reduces body weight, increases total energy expenditure, improves IR in high-fat diet-fed mice by increasing uncoupling protein 3 mRNA expression in skeletal muscle and maintaining PPARγ and adiponectin levels in WAT [200], and promotes adipocyte differentiation, improving basal glucose and insulin-stimulated glucose uptake [201]. It regulates the expression of IL-8 and leukotriene B4 and monocyte migration under the influence of cytokine inducers such as cigarette smoke extraction and LPS [202].

#### 5.1.7. Visfatin

Visfatin is a β-cell differentiation modulator expressed in lymphocytes, bone marrow, muscle, liver [203], and AT (especially visceral AT) [204]. It is a NAD biosynthetic enzyme, the haplodeficiency or chemical inhibition of which cause defects in NAD biosynthesis and glucose-stimulated insulin secretion [205]. Visfatin activates the insulin receptor and ERK1/2 signaling, thus improving glucose uptake in adipocytes and myocytes [206]. However, several studies report that circulating visfatin levels are high in obese individuals and that visfatin is an AT proinflammatory molecule associated with systemic IR and hyperlipidemia [207,208]. Serum visfatin levels have been shown to positively associate with serum IL-6 and *C*-reactive protein but not with IR [209]. The role of visfatin, therefore, is still a debatable issue and requires in-depth experimentation for clarification.

#### 5.1.8. Retinol Binding Protein 4 (RBP4)

RBP4 is a hepatocyte-synthesized protein that aids in vitamin A transport in the body [210]. Additionally, it is secreted by adipocytes that affect insulin sensitivity [211]. RBP4 levels are elevated in insulin-resistant mice and humans with obesity and type 2 DM. The injection of recombinant RBP4 or the transgenic overexpression of human RBP4 in normal mice causes IR [211]. RBP4 is produced by visceral adipose tissue in states of obesity and IR [212]. In insulin-resistant mice, RBP4 expression is high in AT, and AT RBP4 mRNA expression is correlated with serum RBP4 levels [212]. RBP4 inhibits the insulin-induced phosphorylation of IRS-1 and ERK1/2 in primary adipocytes [213]. Apart from markers of obesity and IR, RBP4 is also correlated with inflammatory factors [214].

#### 5.1.9. Angiopoietin-Like 2 (ANGPTL2)

ANGPTL2 is an adipocyte-derived inflammatory mediator promoting adiposity, inflammation and IR, and it is highly expressed in obese states in both mice and humans [215]. Its overexpression in AT promotes inflammation and IR in nonobese mice, whereas its deficiency ameliorates AT inflammation and IR in diet-induced obese mice. It activates integrin signaling to induce an inflammatory cascade and promotes chemotaxis of monocytes and macrophages [215].

#### 5.1.10. Secreted Frizzled-Related Protein 5 (SFRP5)

SFRP5, a protein linked to the Wnt signaling pathway, is an insulin-sensitizing and anti-inflammatory adipokine expressed at higher levels in AT, exhibiting beneficial effects on metabolic dysfunction [216]. Its deficiency in high-calorie diet-fed mice exhibited impaired insulin sensitivity, increased risk for NAFLD and adipose inflammation, and the accumulation of macrophages and proinflammatory cytokines in AT by activating JNK1. Conversely, SFRP5 administration enhanced metabolic function and reduced adipose inflammation in obese states [216]. Circulating SFRP5 was decreased in children with obesity and metabolic syndrome and, upon weight loss, its levels showed an increase [217].

### 5.2. Function of Adipose Tissue-Produced Adipokines in Dyslipidemia

High levels of FFA released from AT through increased lipolysis because of IR are delivered to the liver. The enhanced FFA increases TG and VLDL production in the liver. The inhibition of lipases in AT and skeletal muscle and increased VLDL in the liver inhibiting the lipolysis of chylomicrons contribute to hypertriglyceridemia. The TG in VLDL produces TG-rich LDL and HDL through an exchange for cholesteryl esters from LDL and HDL via cholesteryl ester transport protein. The hepatic lipase hydrolyzes TG in LDL and HDL to both small, dense LDL and HDL. The reduced HDL concentration and formation of small, dense LDL are highly associated with metabolic diseases.

#### Cytokines and Serum Amyloid A (SAA)

TNF-α has been initially recognized to induce hypertriglyceridemia. Hyperlipidemic patients with higher levels of plasma TNF-α are positively correlated with VLDL TG concentration [95], promoting hepatic TG synthesis and secretion [96] and LPL inhibition [97]. Additionally, TNF-α promotes the synthesis of hepatic apolipoprotein (apo) B100-containing VLDL by impairing hepatic insulin signaling [98]. IL-6 is also associated with hypertriglyceridemia and the anti-inflammatory cytokine. IL-10 levels showed an inverse correlation with increased TG, total cholesterol and LDL-cholesterol levels, and metabolic syndrome [101,218]. Cytokines, such as TNF-α, IL-1, and IFN-α, have been reported to stimulate TG synthesis [102] and promote lipolysis in adipocytes [103,104]. The combination of TNF-α and IL-1 increased serum cholesterol, decreased HDL, increased hepatic HMG CoA reductase, and decreased hepatic apo E or apo A–I mRNAs [105]. TNF-α, TGF-β, or IL-1 contribute to lipid deposition and foam cell formation by promoting lipoprotein uptake by the scavenger receptor and the LDL receptor and inhibiting ABCA1-mediated cholesterol efflux to HDL [111,112,113].

SAA proteins, found in the liver and AT and markedly elevated in acute phase response, are expressed highly in obese subjects [219,220]. SAA also play a role in the inflammatory process. SAA treatment increases the expression of IL-6 and TNF-α in preadipocytes and adipocytes [221,222]. Additionally, SAA affect HDL-cholesterol metabolism by inhibiting scavenger receptor SR-BI-mediated HDL binding and selective lipid uptake [223]. SR-BI mediates the cellular uptake of cholesteryl esters from HDL, ultimately promoting the reversal of cholesterol transport from the periphery to the liver [224]. The increased expression of SAA promotes dyslipidemia by affecting HDL structure and function as well as inflammation [225].

### 5.3. Function of AT-Produced Adipokines in NAFLD

The most common form of chronic liver disease is NAFLD [226], and its incidence increases parallelly with a rise in the incidence of obesity [30]. More than two-thirds of NAFLD patients are obese [227]. The two steps of liver injury involved in NAFLD are, firstly, the accumulation of TG in the liver (hepatic steatosis) and, secondly, inflammation and subsequent fibrosis (nonalcoholic steatohepatitis, NASH) [228]. The “two-hit” hypothesis explaining the development of NAFLD and the progression from simple steatosis to NASH is the “first hit,” the accumulation of hepatic lipids, and the “second hit” promotes hepatocyte injury, inflammation, and fibrosis. The second hit is under the influence of proinflammatory cytokines, adipokines, mitochondrial dysfunction, oxidative stress, and subsequent lipid peroxidation [229].

Hyperinsulinemia results in steatosis through increased de novo hepatic lipogenesis and efflux of FFA due to high lipolysis from AT and decreased FFA oxidation and hepatic VLDL secretion. The influx of lipids induces excessive lipid accumulation through high FA import or de novo FA synthesis, overriding hepatic lipid clearance by FA oxidation or the export of TG [230,231]. Insulin failure in suppressing lipolysis results in the secretion of FFA from AT. Elevated lipolysis in AT increases FFA influx into the liver through the portal vein [232]. FFA from enlarged AT are then taken up by the hepatocytes, and a reduction in hepatic insulin clearance further increases circulating insulin levels [233]. The contribution of visceral AT lipolysis to the delivery of FFA is only 5–10% in normal-weight subjects and up to 25% in intra-abdominally obese subjects [234]. After developing steatosis, the liver becomes more prone to “multihit,” such as bacterial toxins, an imbalance in adipokine/cytokine, mitochondrial dysfunction, oxidative damage, impaired hepatocyte apoptosis, profibrogenic factors, and proinflammatory mediators, released from dysregulated organelles and the activated hepatic stellate cell and Kupffer cells. These multiple factors stimulate inflammation, apoptosis, and fibrosis, ultimately leading to progressive liver disease. Macrophage infiltration and the secretion of proinflammatory chemokines, cytokines, and adipokines are promoted by expanded AT, which is closely related to IR [68].

ADP protects the liver from steatosis and inflammation and increases the ability of insulin to suppress glucose production and glucose output [235]. It also inhibits hepatic lipogenesis by suppressing lipogenic transcription factor SREBP1-c [34] and promotes glucose utilization and FA oxidation in the liver by AMPK activation [38]. Circulating ADP levels are low in NAFLD individuals [235] and negatively correlated with liver function markers in healthy individuals [236]. Hepatic steatosis and increased liver injury enzyme levels are predicted by low ADP levels in obese individuals [237]. Additionally, ADP and AdipoR2 expression are reduced in NASH patients [238].

Leptin is another key regulator of NAFLD that stimulates AMPK involved in the activation of lipid oxidation and the inhibition of lipogenesis [239]. However, leptin has distinct roles in different hepatic cells. Leptin function in hepatocytes protects from hepatic steatosis and protects from hepatic injury, whereas it activates HSCs with the production of α-smooth muscle actin, collagen, and TIMP-1, leading to hepatic fibrosis, and upregulates the expression of TGF-β1 in Kupffer cells [240,241,242]. A clinical study demonstrated a positive correlation between circulating leptin and high-serum ALT or hepatic steatosis independent of BMI and body fat, although the significance of this finding is not clear [243].

Among the cytokines, NAFLD patients show increased circulating levels of resistin [244,245], and TNF-α not only mediates the early stages of NAFLD but also transitions to more advanced stages of liver damage [246,247,248]. Acylation-stimulating protein (ASP) and angiotensinogen levels expressed in AT are generally higher in obese subjects [249,250,251]. In NAFLD, the levels of ASP are reported to be associated with IR [249] and angiotensin II antagonists have been shown to improve liver function test and attenuate fibrosis [252].

### 5.4. Role of BCAA as a Mediator of Obesity and NAFLD

Branched-chain amino acids (BCAAs), a group of essential amino acids (leucine, isoleucine, and valine) with nonlinear aliphatic side-chains, are relatively abundant in food, accounting for 20% of total protein intake [253]. They are considered contributors to the development of obesity-associated IR because of the chronic phosphorylation of mTOR, JNK, and IRS1Ser307 and the accumulation of multiple acylcarnitines in muscle [254,255,256]. Levels of fasting plasma BCAAs are found to be elevated in obesity-associated NAFLD [256,257], coinciding with abnormalities in hepatic and AT BCAA catabolic enzymes [258], thus reducing the adipose browning mechanisms. Such malfunctioning in the hepatic BCAA metabolism affects carbon substrate oxidation, impaired antioxidant defense, and ROS generation [254,259]. NAFLD-associated mitochondrial dysfunction (hepatic inflammation) conveys impaired BCAA metabolism, leading to elevated plasma levels of BCAA [260].

A dual effect of BCAA on adipocyte lipolysis and hepatic lipogenesis has been described using HFD and HFD+BCAA mouse models [261]. Although BCAA decreased HFD-induced weight gain and inhibited hepatic lipogenic enzymes, leading to decreased TG content, it induced hepatic damage by activating mTOR, inhibiting hepatic autophagy and increasing hepatic oxidative stress and apoptosis. BCAA increased plasma FFA levels by increasing AMPK-mediated adipocyte lipolysis, and inhibiting lipolysis normalized plasma FFA levels and improved insulin sensitivity but did not protect from BCAA-induced hepatic damage. Thus, BCAA may exacerbate hepatic lipotoxicity [261].

## 6. Therapeutic Implications to Target Adipokines in Obesity-Induced Liver Cancer Progression

The correlation between obesity and cancer is majorly significant because of the convergent pathways involving adipokines, inflammation, and IR. Therapeutic intervention to prevent the effect of obesity on cancer is still under intense scrutiny. The study of bariatric surgery data shows an association between weight loss postsurgery and reduced cancer incidence and metastasis [262]. Patients on insulin or insulin-secreting agents (sulphonylureas) are at higher risks of cancer than patients on oral insulin-sensitizing agents (metformin or thiazolidinediones) [263,264]. Metformin and TZDs have been reported for antitumorigenic effects either as a single agent or in combination with conventional chemotherapeutic agents because of the reduction of insulin and the modulation of intracellular drug targets and epidemiologically both are reported to be associated with low cancer incidence rates [265,266,267]. However, their effect on obese patients with cancer is still not clear. Another potential therapeutic strategy of obesity-induced cancer progression is the chemopreventive modulation of inflammatory pathways.

### 6.1. ADP Agonists

ADP has been identified as a potential therapeutic and preventive agent in altering cancer outcomes, biology, and anticancer immunity. However, the pleiotropic nature of various ADP isoforms poses hurdles in engineering an effective ADP analog [268]. The first ADP receptor agonist with an acceptable safety profile reported was ADP355, which binds both ADP receptors, regulating AMPK, STAT3, PIK3/Akt, and ERK1/2 signaling pathways [268]. ADP355 inhibited the growth of tumor xenografts and protected from CCl_4_-induced liver fibrosis and thioacetamide-induced liver injury [268,269,270,271]. A fluorescence polarization-based screening of 10,000 natural compounds identified AdipoR1 agonists, such as matairesinol, arctiin, (–)-arctigenin, and gramine, as well as AdipoR2 agonists, such as parthenolide, taxifoliol, deoxyschizandrin, and syringin [272]. AdipoRon, an orally active synthetic small molecule AdipoR agonist, which activates both AdipoR1 and AdipoR2, activates AMPK and PPAR-α signaling and ameliorated IR and glucose intolerance in mice fed a HFD [273]. However, these agonists have not been tested for their efficacy in inhibiting obesity-associated liver cancer. In a phase I clinical trial, Efatutazone, a highly selective agonist of PPARγ, in patients with solid cancers showed a dose-dependent increase in plasma ADP levels and delayed invasive progression of mammary carcinogenesis [274,275]. PPARγ agonists, TZDs, induced ADP expression and secretion in a dose- and time-dependent manner in humans and rodents in vitro and in vivo and suppressed the growth, migration, and invasion of cancer cells by augmenting ADP expression and inhibiting leptin signaling [276,277]. As insulin sensitizers, both TZDs and metformin have shown efficacy in rodent models of NASH-HCC and metformin treatment showed a small but significant improvement in survival in patients with type 2 DM and HCC in a clinical study [278,279,280].

### 6.2. Leptin Inhibitors

Leptin exerts pleiotropic functions so that, on the one hand, it increases insulin sensitivity in hepatocytes and, on the other, it promotes hepatic fibrosis via HSCs and can directly stimulate proliferation and antiapoptosis in cancer cells to augment cancer progression. Both leptin and its receptor are overexpressed in HCC. Leptin activates oncogenic signaling pathways, such as PI3K/Akt, MAPK, and STAT3 and upregulates human telomerase reverse transcriptase (hTERT), a mediator of cellular immortalization, in HCC cells [281,282]. Leptin inhibition has been tested as a therapeutic strategy mostly in breast cancer. According to the leptin binding site, leptin peptide receptor antagonist (LPrA) comprises LprA1 and LprA2. LprA2 is conjugated to polyethylene glycol 20kDa (PEG-LprA2) or iron-oxide nanoparticles. PEG-LprA2 inhibits breast cancer by interfering with the expression of proangiogenic factors (VEGF/VEGFR-2) and proproliferative factors (PCNA, cyclin D1) [283]. The in vivo efficacy of PEG-LPrA2 was more significant because of paracrine signaling between stromal, endothelial, and/or inflammatory cells, which play an important role in cancer progression. LPrA2, conjugated with iron-oxide nanoparticles, reduced leptin-induced chemoresistance for cisplatin, cyclophosphamide, paclitaxel, and doxorubicin in breast cancer [284]. LDFI, a leptin receptor antagonist peptide, suppressed the growth of breast cancer cells by inhibiting leptin downstream molecules, such as JAK2, STAT3, AKT, and MAPK [285]. A JAK2 inhibitor AG490 inhibits STAT3 phosphorylation, cell proliferation, HER2 transphosphorylation, and HER2 stabilization by suppressing leptin/Jak2/STAT3 signaling [286,287,288]. Recently, in vitro studies with synthetic leptin derivatives demonstrated inhibition of the proliferation of HCC cells [289]. However, considering the role leptin plays in IR, fibrosis, and HCC, carefully monitored controlled studies with appropriate clinically relevant models need to be performed to use leptin inhibitors as a potential therapy for obesity-induced HCC.

### 6.3. Autotaxin-Lysophosphatidic Acid (ATX-LPA) Signaling Inhibitors

ATX is a secreted enzyme that converts lysophosphatidyl choline into LPA, which signals through LPA receptors to promote hepatic fibrosis and cancer [290]. AT produces ~35% of the body’s ATX, and ATX production is increased during obesity [291]. An autotaxin inhibitor PAT-505 reduced fibrosis in mouse models of NASH and an ATX inhibitor AM063 and an LPAR1 antagonist AM095 decreased fibrosis and reduced HCC development in a diethylnitrosamine (DEN)-induced HCC model [292,293]. Hepatocyte-specific ATX-deficient mice are protected from fibrosis and HCC, thus further establishing the importance of this pathway [294].

### 6.4. IL-6 Inhibitors

Blocking IL-6 signaling might serve as an effective therapeutic strategy for cancer [295]. IL-6 signaling might be inhibited in several ways, such as IL-6-conjugated toxins or IL-6 monoclonal antibodies. A monoclonal antibody, CNTO 328 (siltuximab) inhibits JAK/STAT3 signaling and IL-6/IL-6R/gp130 trans-signaling by interfering with the binding of IL-6 and IL-6R and has shown encouraging results in early trials in ovarian and renal cancers [296,297,298]. Similarly, tocilizumab (MRA) is a humanized antihuman IL-6R antibody that inhibits IL-6 signaling [299]. Based on the importance of IL-6 in regulating NASH and HCC, in principle, the IL-6 blockade might be beneficial in obesity-induced liver cancer. However, as of now, this strategy has not been reported.

A summary of the various avenues as effective therapeutic regimens targeting adipokines is listed in Table 3.

**Table 3:** Adiponectin and adipokines as therapeutic targets in cancer.

Inhibitors	Mechanism	Therapeutic Effect	References
ADP-355	Adiponectin receptor agonist	Suppress tumor growth by binding to AdipoR1 and AdipoR2	[268]
Efatutazone	PPARγ agonist	Delay invasive progression in mammary DCIS and induce differentiation of cancer	[275]
Rosiglitazone	ADPN expression enhancer Leptin signaling inhibitor	Suppress growth, migration, and invasion of cancer cells by inhibiting	[277]
Troglitazone	PPARγ activator	Suppresses growth, migration, and invasion of cancer cells	[276,277]
PEG-LPrA2	Interferes with the proangiogenic factors and proproliferative factors	Inhibit tumor growth	[283]
LDFI	Leptin peptide antagonist binding to the leptin binding site I	Suppresses the growth of cancer cells	[285]
AG490	Suppress leptin/Jak2/STAT3 signaling	Inhibits cell proliferation	[286,287,288]
AM063 and AM095	ATX inhibitor and LPAR1 antagonist, respectively	Protects from hepatic fibrosis and abrogates HCC development	[292,293]
CNTO 328 (siltuximab)	IL-6/IL-6R/gp130 trans-signaling inhibitor	Suppress tumor growth by interfering in the binding of IL-6 and IL-6R	[296,297]
Triacsin C	TAG accumulation into lipid droplets	Inhibits apoptosis by targeting intracellular long-chain acyl-CoA synthetases in NASH	[300]
Isoquercitrin	Activates glucagon-like peptide-1	Targets dipeptidyl peptidase-IV and inhibits apoptotic signaling in NASH	[301]

## 7. Conclusions

**Figure 2:**
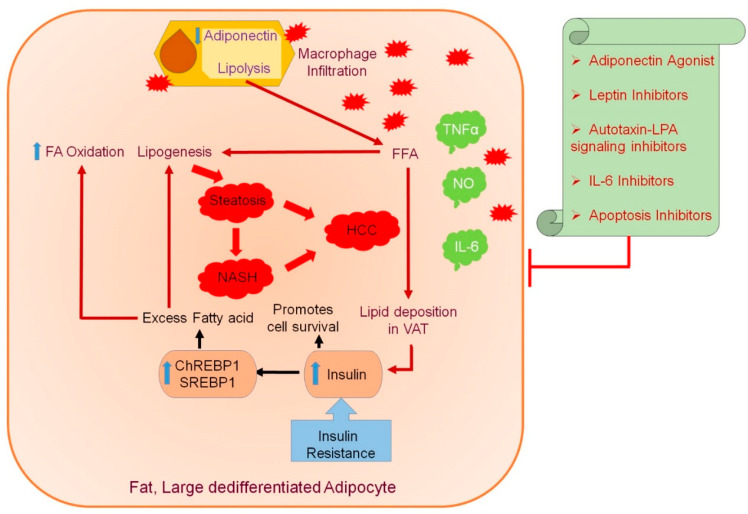
Correlation between adipose tissue and adipokines with the development of obesity-induced hepatocellular carcinoma (HCC) and the effect of adiponectin agonists and other inhibitors. The adipose tissue (AT) secretes various bioactive peptides, known as adipokines, and their associated dysregulation has been implicated in metabolic disorders caused by obesity. The swollen and dedifferentiated adipocytes secrete less adiponectin (ADP) during obesity, and ADP is the only adipokine of which the circulating levels are significantly reduced during obesity. In muscles and the liver, ADP increases insulin action and exerts an antiatherogenic effect. Leptin is another adipokine that regulates energy balance and exerts an insulin-sensitizing effect, but its beneficial effects are reduced in obesity due to leptin resistance. In obese individuals, an inflammatory state is contributed through macrophage infiltration due to excessive production of TNFα, IL-6, or resistin deteriorating insulin action in the liver. Increased lipolysis secretes FAs that accumulate triglyceride in visceral adipose tissue (VAT) in correlation with insulin resistance (IR). The IR and elevated FFAs predispose to steatosis, promoting hepatic lipogenesis and increasing SREBP1 and ChREBP1 expression, further promoting damage to liver tissues and a state of inflammatory response, resulting in NASH followed by HCC. The inflammatory response promoted by obese AT contributes to damage of hepatic cells and impaired immune response. The administration of ADP agonists and AT targeting inhibitors might be a promising therapeutic target for adipokine-mediated obesity-induced HCC. Blue upward arrow indicates an increase and blue downward arrow indicates a decrease.

A multitude of studies place AT as a pivotal regulator of obesity-associated metabolic disorders, such as IR, dyslipidemia, and NAFLD. Enlarged AT leads to macrophage infiltration, and an imbalance between proinflammatory and anti-inflammatory factors secreted by AT leads to inflammation, impaired insulin sensitivity, and the dysregulation of lipid metabolism. Excessive FFA released by AT aids in the initiation and progression of obesity-induced metabolic complications, inducing steatosis and steatohepatitis in the liver, ultimately leading to the development of liver cancer. An unfavorable adipokine profile, induced by obesity, with reduced adipokines having anti-inflammatory or antitumor activity, further paves the way for hepatocarcinogenesis. An imbalanced calorie intake and high insulin levels stimulate lipid accumulation in adipocytes, thus suppressing ADP secretion, leading to an inflammatory state by macrophage infiltration (Figure 2). The subsequent lipolysis leads to the accumulation of triglycerides in visceral adipose tissue (VAT) through secreted FAs, predisposing to steatosis, NASH to HCC. Obese AT promotes the inflammatory response, thus damaging hepatic cells and impairing immune response. These observations open up novel avenues for therapeutic intervention of obesity-associated hepatic malignancies. It will be worthwhile to perform an in-depth exploration of the role of existing ADP agonists, AT, and apoptosis inhibitors administered in other cancers (Table 3) for regulating adipokine-mediated obesity-induced HCC. The correlation between adipose tissue and adipokines with the development of obesity-induced HCC and the probable impact of adiponectin agonists and other inhibitors are summarized in Figure 2.

In summary, the humongous metabolic catastrophe, associated with obesity, creates a major hindrance in the effective implementation of anticancer strategies that have been shown to be effective in cancers in nonobese individuals. In-depth future research will further illuminate the functions and mechanisms of AT-secreted biomolecules for a better understanding of the underlying molecular mechanism of obesity-associated liver cancer. This knowledge will help design appropriate combinatorial therapy to target both the metabolic abnormalities and eliminate malignant cells to effectively combat the disease. The identification of molecules that can regulate both metabolic and oncogenic abnormalities will also help develop effective, targeted therapy. Our studies over the years have documented a critical role of the oncogene astrocyte elevated gene-1 (AEG-1) in regulating both steatosis and inflammation, activating PI3K/Akt signaling and contributing to IR, as well as driving the development and progression of HCC [302,303,304,305]. We documented that the delivery of AEG-1 siRNA using a hepatocyte-targeted nanoparticle could effectively protect from HFD-induced NASH and inhibit orthotopic human HCC xenografts [302,305]. Further comprehensive studies are required to determine the efficacy of this strategy in a model of NASH-HCC and interrogate the role of AEG-1 and similar molecules in regulating AT function.

## Figures and Tables

**Table 1:** List of proteins secreted by adipocytes during normal conditions and hypertrophy.

Proteins	Type
Cytokines	TNF-α, IL-1, IL-6, IL-10
Growth Factor	TGF-β
Metabolites	Leptin, Resistin, Adiponectin
Monocyte Protein	Monocyte Chemoattractant Protein-1 (MCP-1), CXCL5
Hemostatic Proteins	Plasminogen Activator Inhibitor-1 (PAI-1)
Blood-Pressure-Regulating Protein	Angiotensinogen
Angiogenic Proteins	VEGF
